# Investigation of the Positional Relationship Between the Tympanic Segment of the Chorda Tympani and the Incus in Patients With Otosclerosis or Middle Ear Anomalies Using Ultra-High Resolution Computed Tomography

**DOI:** 10.1155/rrp/9486057

**Published:** 2025-02-20

**Authors:** Aya Katsura, Shinsuke Kaneshiro, Harukazu Hiraumi, Makiko Obara, Akio Tamura, Kiyoto Shiga, Kunihiro Yoshioka

**Affiliations:** ^1^Department of Otolaryngology-Head and Neck Surgery, Iwate Medical University, Yahaba, Iwate, Japan; ^2^Department of Otolaryngology-Head and Neck Surgery, Tenri Hospital, Tenri, Nara, Japan; ^3^Department of Radiology, Iwate Medical University, Yahaba, Iwate, Japan

**Keywords:** chorda tympani, computed tomography, imaging, incus

## Abstract

**Objective:** This study evaluated the sensitivity and accuracy of ultra-high-resolution computed tomography (U-HRCT) in predicting the relationship between the chorda tympani and the long crus of the incus in patients with otosclerosis or middle ear anomalies.

**Methods:** Thirteen patients with otosclerosis or middle ear anomalies were enrolled in this study (three males and ten females; mean age, 41.6 years; range, 7–67 years). The patients underwent U-HRCT (Aquilion Precision; Canon Medical Systems, Japan). Multiplanar reconstruction images were obtained, and the distance between the chorda tympani and the long crus of the incus was measured in a plane parallel to the superstructure of the stapes. In addition, the distance between the two structures was measured during surgery. Subsequently, distances of every 0.5 mm obtained using the two modalities were grouped and compared.

**Results:** The U-HRCT-based evaluation revealed that the mean distance from the chorda tympani to the long crus of the incus was 0.7 mm, whereas the mean actual distance during the surgery was 0.9 mm. In nine of the 13 patients, the U-HRCT-based and actual distances belonged to the same group. In four patients, the U-HRCT measurements were smaller than the actual distances. The chorda tympani was attached to the long crus of the incus in three cases, and U-HRCT precisely predicted this finding in all three cases.

**Conclusion:** U-HRCT is useful for detecting the relationship between the chorda tympani and the long crus of the incus in patients with otosclerosis or middle ear anomalies.

## 1. Introduction

The chorda tympani innervates two thirds of the anterior tongue. Damage to this nerve leads to hypogeusia, dysgeusia, metallic taste, mouth dryness, tongue discomfort, and salivation disturbances [1]. The chorda tympani branches from the mastoid portion of the facial nerve and passes through the posterior canaliculus. Within the tympanic cavity, it courses between the malleus handle and the long crus of the incus (tympanic segment). It enters the petrotympanic fissure, medial to the temporomandibular joint (anterior canaliculus). During stapes surgery, a prosthesis is placed on the long crus of the incus. The position of the tympanic segment of the chorda tympani is highly variable and is associated with the risk of surgical injury to the nerve. A chorda tympani close to the incus is at high risk of surgical injury. Preoperative prediction of the relationship between the chorda tympani and the incus is challenging. The chorda tympani within the posterior canaliculus can be visualized using high-resolution computed tomography (CT) and cone-beam CT [2, 3]; however, it is difficult to detect after it exits the posterior canaliculus [3]. Recently, CT with a higher spatial resolution has become available in clinics. Ultra-high-resolution CT (U-HRCT) is a newly developed CT machine with 128 rows of fine detector elements that can detect the chorda tympani within the tympanic cavity [4–6]. However, the accuracy of U-HRCT in determining the distance between the chorda tympani and the incus long process has not been explored. Preoperative evaluation of the distance between the two sites will help in preoperative surgical risk assessment. Selecting the appropriate surgical approach based on this assessment can reduce surgical damage to the chorda tympani.

The present study aimed to evaluate the sensitivity and accuracy of U-HRCT by comparing the distances between the chorda tympani and the long crus of the incus measured using U-HRCT and those calculated using intraoperative findings in patients with otosclerosis or middle ear anomalies.

## 2. Materials and Methods

This was a prospective cohort study. Twenty-six patients underwent stapes surgery for otosclerosis or middle ear anomalies between August 2020 and June 2022 at Iwate Medical University. More than half of the patients underwent conventional CT before arriving at our department, and most did not agree to undergo U-HRCT. Finally, 13 participants were included in the present study (seven ears with otosclerosis, six ears with middle ear anomalies, and three males and 10 females, with an average age of 41.6 years ranging between 7 and 67 years). The patient backgrounds are summarized in Table 1. All participants or their legally authorized representatives provided written informed consent. This study was approved by the Ethics Committee of Iwate Medical University (approval number: MH2020-001) and was conducted in accordance with the Declaration of Helsinki. Images were acquired in a clinical setting using U-HRCT (Aquilion Precision; Canon Medical Systems, Japan). The size of the detector element was 0.25 mm. The acquisition parameters were 120 kV and 190–300 mA (average, 219.3 mA). The reconstruction parameters were as follows: matrix size, 512  ×  512; slice thickness, 0.25 mm; field of view, 90 mm; reconstruction kernel, FC81. U-HRCT covers 40 mm with a single rotation. To reduce total radiation exposure, images were acquired using nonhelical scanning with an acquisition time of 1.5 s. The participants wore glasses to protect their intraocular lenses.

**Figure 1:**
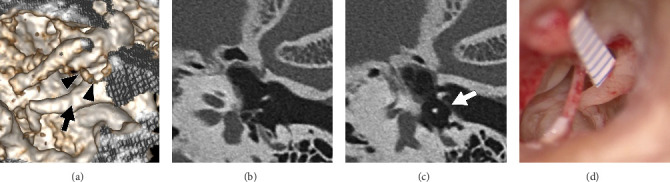
Technique used to evaluate the tympanic segment of the chorda tympani and the long crus of the incus (left ear). (a) A three-dimensional reconstructed image was created, and a linear structure exiting the posterior canaliculus and running between the long crus of the incus and handle of the malleus was judged as the tympanic segment of the chorda tympani. (b) A plane including the superstructure of the stapes was created using multiplanar reconstruction. (c) In a plane parallel to the first plane, including the tympanic segment of the chorda tympani, the distance between the chorda tympani and the long crus of the incus was measured. (d) During surgery, the distance between the two structures was measured. The surgical field was digitally captured, and the distance between the chorda tympani and the long crus of the incus was calculated using ImageJ.

The dose-length product was calculated as 176.5 (104.6–241.4) mGy cm (CT dose index 44.1 [26.2–60.4] mGy). The acquired images were three-dimensionally reconstructed using the RadiAnt DICOM viewer (Medixant, USA). A linear structure exiting the posterior canaliculus and running between the long crus of the incus and the malleus handle was judged as the chorda tympani (Figure 1(a)). The distances between the chorda tympani and the incus long crus were measured using multiplanar reconstruction (MPR) images because the best window level and width for the three-dimensionally reconstructed images differed among patients. A plane that included the superstructure of the stapes was created using the MPR (Figure 1(b)). A new plane, parallel to the first plane, was designed to include the tympanic segment of the chorda tympani (Figure 1(c)). The distance between the chorda tympani and the long crus of the incus was measured in this plane. U-HRCT-based measurements were conducted by a single researcher blinded to the surgical findings (SK). All surgeries were performed under a microscope. The tympanomeatal flap was meticulously elevated to avoid shifting the chorda tympani. No adhesions were observed between the tympanic membrane and chorda tympani. A minimal portion of the posterior bony annulus was removed using a small chisel to preserve the position of the chorda tympani. Direct manipulation of the chorda tympani was avoided during the surgery. No shifts in the chorda tympani were detected under the microscope. Once the chorda tympani and the long crus of the incus were exposed, a measurement device was placed between them. The long crus of the incus was normal in all patients with otosclerosis. In patients with middle ear anomalies, the long crus of the incus was normal, except for the lenticular process (partial defect in two ears and fixation to the stapes in three ears). Deformities of the stapes superstructures were found in four ears (a defect of the posterior crus in two ears and a narrow obturator foramen in two ears). All the surgeries were performed by a single researcher (HH). The surgical field was digitally captured, and the distance between the chorda tympani and the incus long crus was calculated using ImageJ software (NIH, USA) (Figure 1(d)). The distances acquired using both procedures were grouped by 0.5 mm steps (0–0.25, 0.25–0.75, 0.75–1.25, 1.25–1.75, and 1.75–2.25 mm). All digitally captured images were analyzed by a single researcher (AK).

## 3. Results

**Figure 2:**
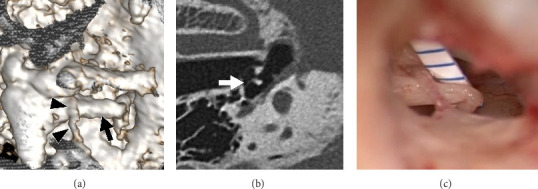
In three patients, the tympanic portion of the chorda tympani was in contact with the incus long crus, as visible in a three-dimensionally reconstructed image (a) and multiplaner reconstruction image (b). In all such cases, the chorda tympani was found to be attached to the incus long crus during the surgery (c).

The tympanic portion of the chorda tympani was detected in all reconstructed three-dimensional images. The tympanic portion of the chorda tympani was separated from the long crus of the incus in 10 patients, whereas it was in contact with the incus long crus in three patients (Figure 2(a)). The distance between the chorda tympani and the long crus of the incus was successfully measured using MPR images. The distance based on U-HRCT was 0.0–1.3 mm (mean: 0.7 mm), while the surgical distance between them was 0.0–2.2 mm (mean: 0.9 mm). In nine of the 13 cases, the distances based on U-HRCT were grouped as identical to the surgical distances. In the other four cases, the distances based on U-HRCT were grouped as shorter than the surgical distances by 0.5–1.0 mm (Table 2). The distances measured using U-HRCT were not overestimated in any of the patients compared with the surgical distances. In addition, based on the intraoperative findings in the three ears (Figure 2(c)), the chorda tympani was attached to the long crus of the incus. All of these cases were precisely predicted using preoperative U-HRCT (Figure 2(b)).

## 4. Discussion

In this study, the distances between the chorda tympani and the long crus of the incus were successfully measured using U-HRCT, and the distances based on U-HRCT were identical to the distances measured during surgery in most cases. In some cases, the distances based on U-HRCT were shorter than the actual distances. Previous CT-based studies reported that the tympanic segment of the chorda tympani is detectable; however, its accuracy has not been examined [4–6]. This is the first study to quantitatively evaluate the accuracy of U-HRCT-based measurements of the tympanic segment of the chorda tympani.

Chorda tympani injury during stapes surgery is a common but underestimated complication. The chorda tympani is damaged during surgery in 7.4%–20.4% of the patients [7, 8]. An injury to the chorda tympani can result in taste discomfort, altered salivation, etc. [3]. Understanding the normal anatomy of the chorda tympani within the tympanic cavity can reduce the risk of iatrogenic injury [6]. In addition, the tympanic segment of the chorda tympani is in contact with or is very close to the surrounding structures in some cases and is at risk of unpredictable iatrogenic complications.

Recently, U-HRCT has been used to visualize the chorda tympani in the tympanic cavity of normal ears [4, 5]. However, these studies used visibility scores and their accuracy has not yet been examined. In the present study, the distance between the chorda tympani and the long crus of the incus was measured using U-HRCT, and the results were compared with the surgical distance. The distance between the two procedures was identical in nine patients, confirming the accuracy of U-HRCT. Importantly, adhesion of the chorda tympani to the incus long crus was precisely predicted using U-HRCT. Since such cases are at a high risk of injury to the chorda tympani, preoperative evaluation with U-HRCT is highly beneficial, especially for those with serious disadvantages such as dysgeusia.

In four cases, the U-HRCT distance was shorter than the surgical distance. This could be due to errors in the surgical measurements. During surgery, the measurement was performed perpendicular to the long crus of the incus, and the distance was corrected using the ImageJ software. However, the three-dimensional angle error cannot be corrected. If the measurements were performed obliquely on the structure, the measured distance would be greater than the actual distance. Another explanation is that the chorda tympani may have shifted during surgery. In the present study, the tympanic membrane was elevated and the posterior bony annulus was removed to visualize the chorda tympani and the long crus of the incus. Meticulous care was taken to not change the position of the chorda tympani during surgery and no positional shifts of the chorda tympani were observed. Despite the precise surgical technique, it is possible that the chorda tympani shifted to elevate the tympanic membrane since it is in contact with the tympanic membrane in some cases [5]. Another explanation could be that the U-HRCT settings were imperfect. The CT images may cause the bone to appear larger than its actual size when the window width is narrow. In the present study, the window level and width were set to 500 and 5500, respectively, to avoid overestimating bone size. However, even under these conditions, the incus may be imaged as more significant than its actual size.

Studies on the clinical classification of the tympanic segment of the chorda tympani are limited. Uranaka et al. proposed a classification system based on the orifices of the posterior canaliculus, tympanic annulus, and incuido-stapedial joint [9]. This classification provides information on the surgical approaches used in endoscopic stapes surgery [10]. However, in this classification, the relationship between the chorda tympani and the long crus of the incus was not considered. This may partly be because conventional CT cannot visualize the tympanic segment of the chorda tympani nerve. Uranaka et al. reported that U-HRCT might assist in predicting the anatomy of the chorda tympani nerve before surgery [9]. The present study suggests that U-HRCT can refine the classification of the tympanic segments of the chorda tympani. Recently, photon-counting CT (PCCT) has become available in clinical settings. In conventional CT, x-rays are converted into visible light. Antiscatter grids are required to avoid the interaction of visible light. In PCCT, x-ray photons are directly converted into electric signals and do not require antiscatter grids, which contributes to reducing the size of the detectors without increasing the radiation dose. The pixel size of the detector of the PCCT system is as low as 0.275 mm [11]. Recent studies have reported that PCCT provides better visualization of temporal bone structures with a lower radiation dose than conventional CT [11, 12]. The visibility and accuracy of the chorda tympani were not examined in these studies, but it is possible that PCCT provides comparative accuracy for the chorda tympani with U-HRCT with a reduced radiation dose.

A limitation of the present study is its small sample size. Most patients with otosclerosis and middle ear anomalies undergo conventional CT before arrival at the hospital. In such patients, U-HRCT can be unnecessarily invasive, resulting in only a few patients being enrolled in the study. Another limitation is that the results of the present study cannot be generalized to other middle ear pathologies. In the present U-HRCT setting, the contrast resolution of soft tissue was low, and it may be difficult to detect the chorda tympani in ears with middle ear pathologies such as chronic otitis media and cholesteatoma. Nevertheless, the present study shows that U-HRCT provides additional information to avoid surgical complications in patients with otosclerosis and middle ear anomalies. It may be acceptable to recommend U-HRCT for patients who have undergone conventional CT.

## 5. Conclusions

U-HRCT with multiplanar reconstructed images helps detect the course of the tympanic portion of the chorda tympani and evaluate its relationship with the long crus of the incus. This distance is sufficiently accurate for clinical use in patients with otosclerosis or middle ear anomalies.

## Figures and Tables

**Table 1:** Patient demographics.

Sex	Age	Disease	Side	Distance on U-HRCT (mm)	Actual distance (mm)	Error (mm)
M	52	Otosclerosis	L	0	0	0
F	51	Otosclerosis	L	1.5	2	0.5
M	23	Otosclerosis	L	1	1	0
F	56	Otosclerosis	L	0.5	0.5	0
F	67	Otosclerosis	L	0.5	1.5	1
F	57	Otosclerosis	R	0	0	0
F	50	Otosclerosis	L	0	0	0
M	7	Malformation	R	1	1	0
F	56	Malformation	R	1	2	1
F	36	Malformation	L	1	1	0
F	22	Malformation	L	1	1	0
F	54	Malformation	R	0.5	1	0.5
F	10	Malformation	L	0.5	0.5	0

**Table 2:** Number of patients according to the distance between the chorda tympani and the incus measured on U-HRCT and during the surgery.

Distance measured on U-HRCT (mm)	Actual distance (mm)				
	0	0.5	1	1.5	2
0	3				
0.5		2	1	1	
1			4		1
1.5					1
2					

## Data Availability

The CT data used to support the findings of this study are available from the corresponding author upon reasonable request.
